# Metabolic profiles of captive Asian elephants (*Elephas maximus*) in Lao PDR and Thailand

**DOI:** 10.1371/journal.pone.0334550

**Published:** 2025-12-17

**Authors:** Ana Belén López Pérez, Janine L. Brown, Veerasak Punyapornwithaya, Chatchote Thitaram, Jakkawat Pongsumpan, Jarawee Supanta, Wuithipong Tocharoennirattisai, Jaruwan Khonmee

**Affiliations:** 1 Faculty of Veterinary Medicine, Chiang Mai University, Chiang Mai, Thailand; 2 Elephant, Wildlife and Companion Animals Research Group, Chiang Mai University, Chiang Mai, Thailand; 3 Elephant Conservation Center, Sayaboury, Lao PDR; 4 Smithsonian National Zoo & Conservation Biology Institute, Front Royal, Virginia, United States of America; 5 Akkhraratchakumari Veterinary College, Walailak University, Nakhon Si Thammarat, Thailand; Jimma University College of Agriculture and Veterinary Medicine, ETHIOPIA

## Abstract

This study compared metabolic health biomarkers, lipid profiles, body condition scores (BCS), and adrenocortical function of 78 captive Asian elephants (*Elephas maximus*) managed under contrasting systems in Laos and Thailand. In Laos, elephants are primarily managed in naturalistic forest environments with limited tourist interaction, while many Thai elephants participate in tourism activities like riding and are fed calorie-dense supplemental foods, with restricted access to natural forage. Serum and fecal samples were collected for 1 year to assess serum glucose, insulin, cholesterol, triglycerides, lipoproteins, fecal glucocorticoid metabolites (fGCM), and BCS across seasons, sexes, and management regimens. Thai elephants exhibited significantly higher glucose, insulin, lipid levels, and BCS, reflecting the effects of high-calorie diets and reduced exercise, while Lao elephants displayed healthier metabolic profiles and BCS, attributed to natural foraging and physical activity. In Thai elephants, BCS was positively correlated with insulin, and negatively correlated with the glucose-to-insulin ratio, total cholesterol, HDL, and LDL, suggesting overweight elephants in Thailand are at risk of metabolic derangements. None of those parameters were significantly correlated with BCS in the Laotian population. A surprising finding was higher fGCM in Lao elephants, which could have been related to new social opportunities and pandemic-driven management disruptions at the Elephant Conservation Center. These findings underscore how management practices, particularly diet composition, exercise opportunities, and degree of naturalistic living, can influence the health and welfare of captive elephants and support the adoption of more naturalistic management strategies to improve elephant welfare in captivity.

## Introduction

Elephants are a keystone species with significant impacts on ecosystems and the species therein [1,2]. Despite their ecological importance, wild Asian elephant *(Elephas maximus)* populations continue to decline due to habitat loss and habitat fragmentation, human-elephant conflict, and poaching for body parts such as ivory or skin. The total captive Asian elephant population is approximately 15,000 individuals, corresponding to about one-third of the 45,000 wild Asian elephant population [3]. Maintenance of genetically healthy captive elephant populations can support conservation strategies, such as reintroduction into viable habitats, genetic refreshment of small isolated populations, and research into factors impacting fitness and survival *in situ* and *ex situ* [4–6]. Unfortunately, most captive Asian elephant populations are not self-sustaining due to higher mortality than birth rates [7–9].

Captive elephant numbers are estimated to be ~ 3,800 in Thailand [10] and ~500 in Laos (formally the Lao People’s Democratic Republic) [11]. In Thailand, the majority (about 60%) are found in the north and northeast, primarily in Chiang Mai province [12]. In this region, elephants participate in tourist activities, ranging from hands-off opportunities like observation from afar to more interactive experiences such as feeding, bathing, walking alongside, riding with a saddle or bareback, and elephant shows [13]. Most facilities offer only limited access to forests for foraging or free-roaming, so camp owners design much of the captive diet, which includes mostly farm-grown grasses, like napier grass (*Pennisetum purpureum*), bana grass (P. *purpureum x P. americanum*), sweet corn (*Zea mays var. saccharate*) stover, as well as supplements consisting of fruits and sugar cane [14–17]. In Laos, the majority of captive elephants are in Sayaboury province (~80%) [18]. After the 2016 ban on the export of logs and sawn wood [19], many elephants in Sayaboury were left in the forest without employment, or worked in small-scale logging operations, where they had a natural forage diet [20]. Soon thereafter, some of these elephants began participating in tourist activities, ranging from hands-off opportunities to more traditional activities such as feeding, riding, and elephant shows. Today, elephant-based tourism has become a principal component of the national tourism industry in the country, mostly located in Luang Prabang Province [21].

Across western zoos and Asian tourism settings, management practices can strongly affect elephant health and welfare. [8]. Consequently, there has been an increase in studies to assess how management factors impact elephant health and welfare [14,16,22,23], with considerable agreement between countries [8]. Of particular relevance is the relationship between diet and exercise. In Thailand, high-energy foods (bananas and sugar cane) fed by tourists are associated with high body conditions scores (BCS) and alterations in total cholesterol (TC), low-density lipoproteins (LDL), high-density lipoproteins (HDL), triglycerides (TG), insulin, glucose, fructosamine, and the ratio of glucose to insulin (G:I) [15]. Both rates of obesity and concentrations of fecal glucocorticoid metabolites (fGCM) are lower in elephants with more physical activity [8,15].

In Thailand, noted changes in management practices based on those results were subsequently observed by veterinarians in the Elephant, Wildlife and Companion Animals Research Group, Chiang Mai University, who visit camps regularly. But in March-April 2020, the Thai and Laos governments banned all international travel due to the COVID-19 pandemic [24,25], resulting in the closure of all tourist camps through 2021. Studies published by Supanta et al. [12,13,26] showed that the lack of tourists had significant impacts on tourist camp management and elephant health and welfare in Thailand, in the form of reduced exercise opportunities and food provided, resulting in altered stress biomarkers [26], muscle function, and liver function compared with previous years [12]. However, some benefits were also observed, such as healthier BCS and decreases in lipid markers (TC, TG, LDL) from the lack of tourists feeding sweet foods [12].

While considerable physiological data now exist on tourist elephants in Thailand – both pre- and post-COVID-19 – no studies have examined metabolic or stress markers in elephants managed under more naturalistic conditions, like those in Laos. The objective of this study was to compare metabolic and lipid markers, BCS, and fGCM concentrations between captive elephants in Laos and Thailand to evaluate how differing management approaches impact health and welfare across captivity scenarios. These findings are critical for formulating science-based recommendations, identifying areas for improvement in tourist camps to meet elephants’ fundamental health and welfare needs, and understanding the physiological effects of contrasting management strategies. Though not intentional, data collection took place during the COVID-19 travel ban in both countries. Thus, this natural experiment provided a unique opportunity to assess the direct and indirect effects of captive management practices, with the enforced pause in tourist activities providing a way to disentangle the influence of visitor interactions from other variables.

## Materials and methods

### Study sites

#### Laos.

A total of 27 healthy captive Asian elephants, including 10 males (29.0 ± 16.1 years of age, range, 7–60 years) and 17 females (40.0 ± 8.8 years of age, range, 25–57 years) were included in this study (Table 1). From this population, 19 individuals were housed at the Elephant Conservation Center (ECC) in Sayaboury Province. The ECC consists of 530 hectares of mixed deciduous and dry dipterocarp forest. It is open to the public all year, but tourist activities are limited to remote observations only. Elephants spend the morning (0900–1200 hours) and afternoon (1330–1630 hours) socializing and foraging in different natural areas throughout the venue. At night (1800–0800 hours), elephants are restrained with a 40 m chain within different locations of the forest to allow natural foraging and prevent any conflict with surrounding farmers. The remaining eight elephants were located in the Nam Phouy National Protected Area (Nam Phouy NPA), which covers 191,200 hectares of forest [27] and shelters the second largest population of wild elephants in Laos [28]. Of these, four were part of a soft-release reintroduction program into the protected area with complete freedom to roam (‘released group’), unless intervention was deemed necessary for safety reasons – either to protect the elephants themselves or nearby communities. Once a month, physical health was evaluated by a veterinarian. Four other elephants were housed at Nam Phouy NPA, where mahouts (elephant keeper) employed traditional management techniques involving periodic release into the forest (‘traditional management group’). During the dry season, elephants were alternately tethered on 30–40 m chains or released with a dragging chain in wooded areas near the village. When tethered, they were relocated daily to refresh the feeding grounds and provide access to water. In the rainy season, when human resources were primarily allocated to planting crops, elephants were predominantly released into the forest for extended periods, monitored by mahouts through weekly searches.

**Table 1:** Summary of parameters related to the management of elephants at captive venues in Laos and Thailand.

	Laos			Thailand				
**Location**	Nam Phouy NPA^1^ (released)	Nam Phouy NPA^2^ (traditional management)	ECC	A	B	C	D	E
**Males**	1	0	9	5	2	4	0	0
**Females**	3	4	10	9	8	7	8	8
**Age (years)**	7-48	32-45	9-60	22-50	23-53	19- 45	28-54	30-56
**Blood sample collection**	Monthly	Twice a month	Twice a month	Monthly	Monthly	Monthly	Monthly	Monthly
**Fecal sample collection**	Twice a month	Twice a month	Twice a month	Monthly	Monthly	Monthly	Monthly	Monthly
**Diet**	A wide variety of natural plants, fruits, barks, and roots are available (up to 114 plant species are commonly consumed by the elephants in the landscape) including bamboo species such as *Bambusa tulda*, *Dendrocalamus strictus*, and *Gigantochloa albociliata*; ginger species including *Zingiber zerumbet* and *Alpinia galanga*; banana plants (*Musa spp*.); and grasses like *Imperata cylindrica* and *Pennisetum purpureum.*		Access to natural food sources, including fruits, bark, roots, and a wide variety of plants, is available throughout the year. However, large seasonal changes affect availability: food is abundant during the wettest months (June, July, and August) but limited during the driest months (March, April, and May).	Napier grass, bana grass, and corn stalk (172.86 ± 2.23 kg/day).	Napier grass, bana grass, and corn stalk (131.90 ± 2.70 kg/day).	Napier grass, bana grass, and corn stalk (162.50 ± 2.97 kg/day).	Napier grass, bana grass, and corn stalk (123.96 ± 1.41 kg/day).	Napier grass, bana grass, and corn stalk, (168.55 ± 2.44 kg/day).
**Supplements**^**4**^ **(kg/day)**	0	0	0	9.30 ± 0.68	15.25 ± 1.15	13.88 ± 1.27	14.90 ± 0.99	12.64 ± 0.65
**Walk distance (km/day)**	0.5-8.0	0.5-5.0	2.0-4.0	0.0-1.5	0.0-6.0	1.0-6.0	0.5-2.0	0.5-3.0
**fGCM (ng/g)**	49.01 ± 2.71	49.89 ± 2.03	58.17 ± 1.27	50.03 ± 1.97	57.59 ± 1.98	44.94 ± 1.65	53.53 ± 2.17	46.45 ± 1.78

^1^Captive elephants from the Elephant Conservation Center (ECC) released into the Nam Phouy National Protected Area (Nam Phouy NPA) in 2019 as part of a soft-release program.

^2^Captive elephants living within the Nam Pouy NPA under traditional captive management.

^3^Based on interview surveys from April 2020 to April 2021 published by Supanta et al.2023 [13].

^4^Primarily bananas and sugar cane fed by mahouts/camp owners.

#### Thailand.

A total of 51 healthy elephants from five tourist camps in the Chiang Mai area were involved in a welfare study conducted between 2020 and 2022 in Thailand and provided data on husbandry practices [13] and physiology [12,26] for this study (Table 1). The study population included 11 males (34.5 ± 12.5 years of age, range, 19–56 years) and 40 females (38.0 ± 9.2 years of age, range, 20–56 years). Before the travel ban, elephants were involved in riding with a saddle (Camps B, C, and E) or bareback (Camp B), elephant shows (Camps B and C), bathing with tourists (Camp A), and tourist feeding (all camps). After the lockdown, all tourist activities ceased, leading to significant changes in the care and management of captive elephants. With the absence of visitors, most camps suspended elephant-related activities, resulting in a substantial decline in physical exercise. The average daily walking distance decreased from approximately 4 km to just 0–1 km, and only 47% of camps continued to provide any walking opportunities.

As work activities diminished, chaining time increased. In 77% of camps, elephants were chained for more than 21 hours per day, typically under covered sheds or in woodland areas. Additionally, most camps experienced a notable reduction in the number of employed mahouts, further impacting daily care. Elephant diets were also affected. While the types of roughage provided remained largely the same, the overall quantity of feed was gradually reduced over time [13].

### Sample collection

Blood and fecal samples were collected once or twice a month from February 2020 to April 2021 (Table 1). All samples from Lao elephants were collected and analyzed specifically for this study. In Thailand, comparative values were drawn from previously published data using comparable methodology [12,13,26]. Notably, both countries were under international travel bans due to COVID-19 from early 2020 through at least 2021; thus, samples represent data collected during the pandemic, when tourist activities were limited or absent. 

### Body condition scoring

Body condition was assessed monthly by scoring the back, ribs, and pelvic bone areas using a standardized photographic guide with detailed instructions [29]. Scores ranged from 1 (thinnest) to 5 (fattest), and assessments were made in 0.5-point increments for greater precision [15].

### Serum glucose, insulin, G:I

Serum glucose was measured by automated standardized liquid chromatography-mass spectrometry at the International Medical Diagnostic Center in Vientiane, Laos (Roche Cobas C 311; Cobas® Mass Spec) or an automated glucose analyzer (Glucinet T01-149, Bayer, Barcelona, Spain) at Chiang Mai University in Thailand [12]. Serum insulin concentrations were measured by a solid-phase two-site bovine insulin enzyme immunoassay (EIA; Cat. No. 10−1,113−01; Mercodia, Uppsala, Sweden) validated for elephants [12,23]. Colorimetric responses were determined by a spectrophotometer at 450 nm filter with an Opsys MR Microplate Reader (TECAN Sunrise™ microplate reader; Salzburg, Austria) [12]. All samples were analyzed in duplicate; intra- and inter-assay coefficients of variation were <10 and <15%, respectively.

### Lipid profiles

Serum TC, LDL, HDL, and TG were quantified by automated, standardized liquid chromatography-mass spectrometry Roche Cobas C311 (Cobas® Mass Spec) in Laos and an Automated Clinical Chemistry Analyzer (Sysmex; BX-3010, Sysmex Corporation of Japan) in Thailand [12].

### Hormone processing and extraction

Fecal samples were extracted as described by Norkaew et al. (2018) [15]. Briefly, frozen fresh feces were thawed at room temperature (RT) and dried in a conventional oven at 60°C for ~24−48 hours and stored at −20°C until extraction. Mixed dry powdered feces (~0.1 g ± 0.01) were extracted in 5 mL 90% ethanol by boiling in a water bath (96°C) for 20 minutes and adding 95% ethanol as needed to keep from boiling dry. Samples were centrifuged at 2,500 x g for 20 minutes. Supernatants were transferred into a new glass vial, and the fecal pellets were centrifuged again with 5 mL 90% ethanol. The combined supernatants were dried under air in a 50°C water bath. Dried extracts were reconstituted in 1 mL assay buffer (0.0137 M Trizma base, 0.2 M Tris-HCl, 0.2 M NaCl, 0.2 M EDTA, 0.001% BSA, and 0.001% Tween 20; pH 7.5) and stored at –20°C until enzyme immunoassay (EIA) analysis.

### Hormone analyses

Concentrations of fGCM were determined using a double-antibody EIA with a polyclonal rabbit anti-corticosterone antibody (CJM006) validated for Asian elephants [30] and described by Norkaew et al. (2018) [15]. The absorbance was measured at 450 nm by a microplate reader (TECAN Sunrise™ microplate reader; Salzburg, Austria). Assay sensitivity (based on 90% binding) was 0.14 ng/mL. Samples were diluted 1:4 for female elephant samples and 1:6–1:8 for male elephant samples in assay buffer for analysis. The inter-assay CV for high (30% binding) and low (70% binding) control samples was < 15%. Samples were reanalyzed if the duplicate CV was > 10%; thus, intra-assay CVs were <10%. Fecal data are expressed as ng/g dried feces.

### Statistical analyses

Statistical analyses were performed using R version 4.1.2 [31]. Physiological measurements were collected monthly from most elephants, although some individuals were sampled twice within the same month. In those cases, the two values were averaged, so that each elephant contributed only one data point per month for each parameter. Repeated measures data were analyzed using Generalized Estimating Equations (GEE) to evaluate the effects of location and country, season, and sex on metabolic and lipid parameters, BCS, and fGCM concentrations. The analysis specifically examined: (1) the country effect; (2) the effect of management practices across the three main study locations (ECC, release/traditional management, and Thai tourist camps); (3) the influence of season (summer, winter, and rainy); and (4) the effect of sex on the outcome measures. To test whether seasonal effects differed by country, an interaction term of season and country was incorporated into the model. To assess sex differences, a separate model was fitted with a country and sex interaction. Individuals were treated as a random effect. GEE was fitted to marginal regression models using the geeglm() function from the geepack package in R, specifying an autoregressive correlation structure of order 1 AR (1) to account for within-subject correlation over time [32]. GEE models were followed by Bonferroni’s multiple comparisons of estimated marginal means post-hoc tests with p- value correction. The significance level for all statistical analyses was set at α = 0.05. Associations between BCS and metabolic hormones, lipid profiles, and fGCM, were further evaluated using aggregated data. For each physiological category, all parameters were consolidated, resulting in a single representative value per individual per category for the correlation analysis. Given the ordinal nature of BCS data and the presence of tied values, Kendall’s tau-b correlation coefficient was calculated using the cor.test() function in R (method = “kendall). To visualize the correlation trends, the geom_smooth() function from the ggplot2 package was also applied.

## Results

A total of 1328 serum samples (Laos = 682 samples; Thailand = 646) and 1402 fecal samples (Laos = 806 samples; Thailand = 596) were evaluated in this study. Descriptive metabolic markers, lipid profile, BCS, and fGCM measures for elephants in Laos and Thailand are presented in Table 2. Insulin (β = –0.32, SE = 0.08, Z = –4.16, p < 0.0001), glucose (β = – 12.80, SE = 2.62, Z = –4.88, p < 0.0001), TC (β = –4.42, SE = 2.02, Z = –2.19, p = 0.029), HDL (β = –2.04, SE = 0.56, Z = –3.66, p = 0.0002), LDL (β = –4.93 SE = 1.40, Z = –3.53, p = 0.0004), BCS (β = –0.77, SE = 0.10, Z = –7.43, p < 0.0001) were significantly higher in Thai elephants compared to the Lao population. In contrast, fGCM concentrations were higher in the Lao elephants (β = –5.55, SE = 2.27, Z = –2.44, p = 0.014).

**Table 2:** Country effect on physiological parameters in captive Asian elephants. Mean (± SEM), and range (min-max) values in metabolic factors, lipid panel measures, body condition scores, and fecal glucocorticoid metabolite concentrations in captive Asian elephants in Laos (n = 27) and Thailand (n = 51).

Factors	Country	
	Laos	Thailand
**Insulin (ng/mL)**	0.25 ± 0.02^a^	0.58 ± 0.04^b^
	(0.02-3.26)	(0.02-9.77)
**Glucose (mg/dL)**	75.16 ± 0.73^a^	88.76 ± 1.00^b^
	(32.00-161.00)	(23.00-203.00)
**G:I**	793.79 ± 30.48^a^	740.12 ± 35.15^a^
	(48.63-3840.00)	(10.95-3960.00)
**TC (mg/dL)**	41.69 ± 0.42^a^	46.22 ± 0.57^b^
	(19.33-78.00)	(19.00-124.00)
**TG (mg/dL)**	23.56 ± 0.59^a^	24.80 ± 0.50^a^
	(4.00-122.00)	(2.00-100.00)
**HDL (mg/dL)**	10.51 ± 0.15^a^	12.24 ± 0.15^b^
	(4.00-20.50)	(4.80-33.70)
**LDL (mg/dL)**	25.45 ± 0.29^a^	30.69 ± 0.40^b^
	(9.00-44.50)	(10.30-80.80)
**BCS (1–5)**	2.93 ± 0.02^a^	3.82 ± 0.03^b^
	(2.00-4.00)	(1.50-5.00)
**fGCM (ng/g)**	56.07 ± 1.06^b^	50.40 ± 0.88^a^
	(4.94-161.17)	(16.94-175.96)

Abbreviations: G:I = glucose to insulin ratio; TC = total cholesterol; TG = triglycerides; HDL = high density lipoproteins; LDL = low density lipoproteins; BCS = body condition score; fGCM = fecal glucocorticoid metabolites.

a,b Different superscript letters within columns indicate significant differences for each variable based on GEE model results (p < 0.05). Superscript letters (a, b) were assigned in order of concentration, with a representing the lowest concentration.

Table 3 shows differences in metabolic markers between released/traditionally managed elephants living within the Nam Phouy NPA, the ECC in Laos, and several tourist camps in Thailand (Camps A-E). Significant differences were observed across groups, with insulin, glucose, HDL, and LDL being significantly higher for the Thai elephant compared with the release/traditional management and the ECC elephants (p < 0.05) (S1 Table). BCS was the lowest, and fGCM concentration the highest at ECC among the three groups. Finally, tourist camp elephants in Thailand had significantly higher TC compared with the release/traditional management elephants (p < 0.05).

**Table 3:** Location effect on physiological parameters in captive Asian elephants. Mean (± SEM) and range (min-max) values on metabolic factors, lipid panel measures, body condition scores, and fecal glucocorticoid metabolites for elephants at the Elephant Conservation Center in Laos (n = 19), those released and traditionally managed in Laos (n = 8), and in tourist camps in Thailand (n = 51).

Parameter	Mean ± SEM	Range
**Insulin (ng/mL)**		
ECC	0.27 ± 0.02^a^	0.02-3.26
Released/traditional management^1^	0.19 ± 0.01^a^	0.02-0.58
Thailand tourist camps	0.58 ± 0.04^b^	0.02-9.77
**Glucose (mg/dL)**		
ECC	76.23 ± 0.92^a^	32.00-161.00
Released/traditional management	71.98 ± 0.91^a^	49.00-96.00
Thailand tourist camps	88.76 ± 1.00^b^	23.00-203.00
**G:I**		
ECC	853.28 ± 36.75^a^	48.63-3840.00
Released/traditional management	616.46 ± 48.48^a^	129.07-2520.00
Thailand tourist camps	740.12 ± 35.10^a^	10.95-3960.00
**TC (mg/dL)**		
ECC	42.51 ± 0.48^ab^	20.50-78.00
Released/traditional management	39.24 ± 0.84^a^	19.33-56.00
Thailand tourist camps	46.22 ± 0.57^b^	19.00-124.00
**TG (mg/dL)**		
ECC	21.54 ± 0.47^a^	4.00-57.00
Released/traditional management	29.61 ± 1.75^a^	8.67-122.00
Thailand tourist camps	24.80 ± 0.50^a^	2.00-100.00
**HDL (mg/dL)**		
ECC	11.09 ± 0.17^a^	5.00-20.50
Released/traditional management	8.77 ± 0.28^a^	4.00-16.50
Thailand tourist camps	12.24 ± 0.15^b^	4.80-33.70
**LDL (mg/dL)**		
ECC	26.02 ± 0.35^a^	9.00-44.50
Released/traditional management	23.85 ± 0.46^a^	10.67-35.00
Thailand tourist camps	30.69 ± 0.40^b^	10.30-80.80
**BCS (1–5)**		
ECC	2.79 ± 0.02^a^	2.00-3.50
Released/traditional management	3.35 ± 0.03^b^	3.00-4.00
Thailand tourist camps	3.82 ± 0.03^c^	1.50-5.00
**fGCM (ng/g)**		
ECC	58.17 ± 1.27^b^	4.94-161.17
Released/traditional management	49.45 ± 1.68^a^	13.11-121.05
Thailand tourist camps	50.40 ± 0.88^a^	16.94-175.96

^1^Elephants living in the Nam Phouy National Protected Area: four from the Elephant Conservation (ECC) that were part of a soft-release program in 2019; four managed under traditional conditions.

Abbreviations: G:I = glucose to insulin ratio; TC = total cholesterol; TG = triglycerides; HDL = high density lipoproteins; LDL = low density lipoproteins; BCS = body condition score; fGCM = fecal glucocorticoid metabolites.

^a,b,c^Different superscript letters within locations indicate statistically significant differences between values, based on GEE model results (p < 0.05). Superscript letters (a, b,c) were assigned in order of concentration, with a representing the lowest concentration.

**Fig 1:**
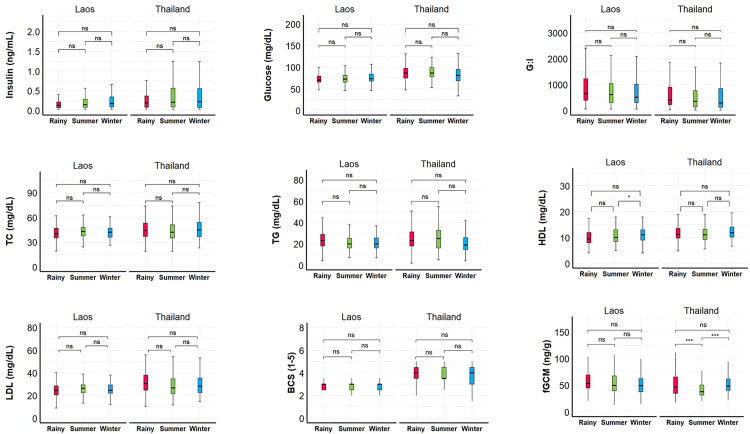
Seasonal effects on physiological parameters in Asian elephants in Laos (n = 27) and tourist camps in Thailand (n = 51). G:I = glucose to insulin ratio; TC = total cholesterol; TG = triglycerides; HDL = high density lipoproteins; LDL = low density lipoproteins; BCS = body condition score; fGCM = fecal glucocorticoid metabolites. Summer = 16 February-15 May; Rainy = 16 May-15 October; Winter = 16 October-15 February. Asterisks above comparisons denote statistical significance as determined by the GEE model: *p < 0.05; **p < 0.01; ***p < 0.001; ns = not significant differences.

Seasonal effects in Laos and Thailand are summarized in Fig 1 (S2 Table). Overall, none of the parameters appeared to be affected by season within each country, with the exception of fGCM concentrations in Thailand, were the lowest concentrations were found in summer compared to rainy and winter seasons (rainy-summer: β = –8.15, SE = 2.05, Z = 3.98, p < 0.0001; summer-winter: β = –10.10, SE = 1.62, Z = –6.23, p < 0.0001), and HDL concentration in Laos, were concentrations in winter differed significantly from summer (β = 0.58, SE = 0.24, Z = 2.45, p = 0.043).

**Fig 2:**
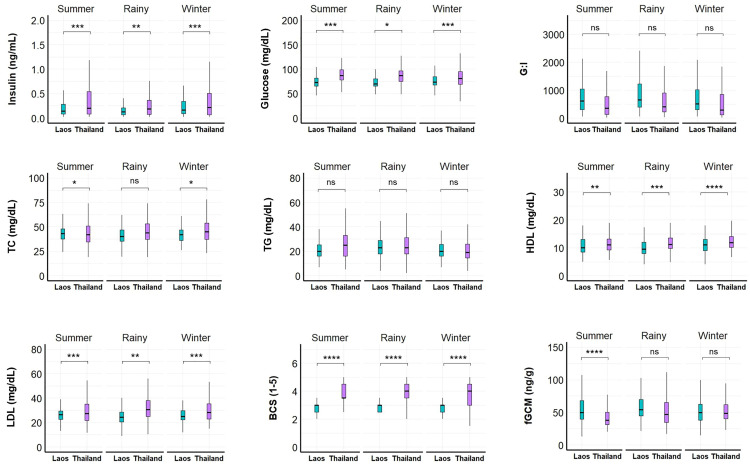
Seasonal differences in physiological parameters between Asian elephants in Laos (n = 27) and tourist camps in Thailand (n = 51). G:I = glucose to insulin ratio; TC = total cholesterol; TG = triglycerides, HDL = high density lipoproteins; LDL = low density lipoproteins; BCS = body condition score; fGCM = fecal glucocorticoid metabolites. Summer = 16 February-15 May; Rainy = 16 May-15 October; Winter = 16 October-15 February. Asterisks denote statistical significance as determined by the GEE model: *p < 0.05; **p < 0.01; ***p < 0.001; ****p < 0.0001; ns = not significant.

Between countries, insulin, glucose, HDL, LDL, and BCS were consistently higher across all seasons in Thailand compared to Laos (Fig 2, S2 and S3 Tables). In addition, TC was higher in summer and winter in Thailand compared to Laos (p < 0.05), and fGCM was higher in summer in Laos compared to Thailand.

**Fig 3:**
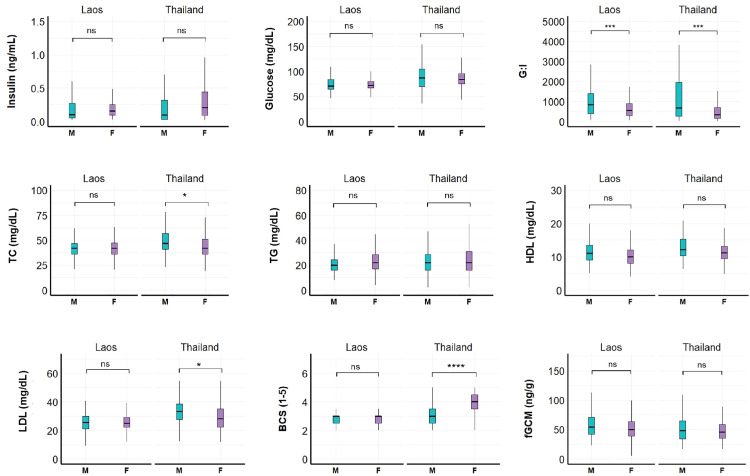
Sex differences in physiological parameters in Asian elephants in Laos (n = 27) and tourist camps in Thailand (n = 51). G:I = glucose to insulin ratio; TC = total cholesterol; TG = triglycerides, HDL = high density lipoproteins; LDL = low density lipoproteins; BCS = body condition score; fGCM = fecal glucocorticoid metabolites. Asterisks denote statistical significance as determined by the GEE model: *p < 0.05; **p < 0.01; ***p < 0.001; ****p < 0.0001; ns = not significant.

Sex differences were observed within and between countries. In Laos, males had higher G:I (β = 368.00, SE = 105.00, Z = 3.52, p = 0.0004) than females (Fig 3, S4 Table). In Thailand, males also had higher G:I (β = 493.00, SE = 142.00, Z = 3.49, p = 0.0005) than females, as well as TC (β = 9.85, SE = 4.19, Z = 2.35, p = 0.02) and LDL (β = 6.12, SE = 2.81, Z = 2.18, p = 0.03), while females had higher BCS (β = –0.94, SE = 0.12, Z = –7.49, p < 0.0001).

**Fig 4:**
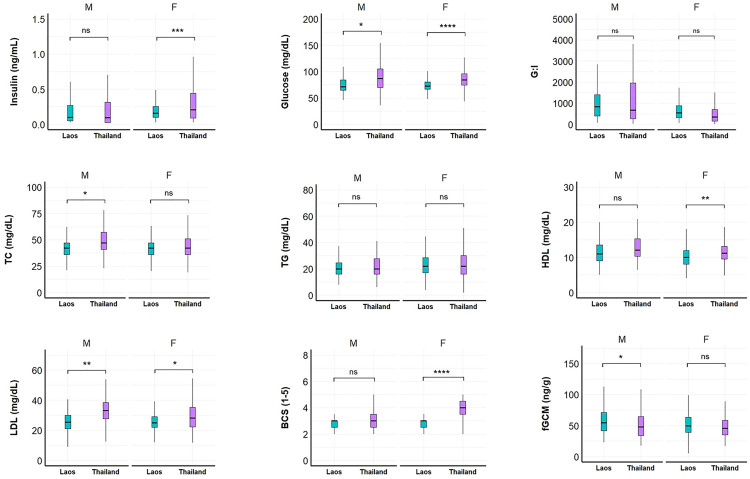
Sex differences in physiological parameters of Asian elephants between Laos (n = 27) and tourist camps in Thailand (n = 51). G:I = glucose to insulin ratio; TC = total cholesterol; TG = triglycerides; LDL = low density lipoproteins; HDL = high density lipoproteins; BCS = body condition score; fGCM = fecal glucocorticoid metabolites. Asterisks denote statistical significance as determined by the GEE model: *p < 0.05; **p < 0.01; ***p < 0.001; ns = not significant.

Among males, Lao elephants had higher fGCM concentrations (β = –4.44, SE = 2.95, Z = –1.50, p = 0.01), while Thai males had higher glucose (β = −14.90, SE = 6.61, Z = –2.25, p = 0.02), TC (β = –12.10, SE = 5.05, Z = –2.39, p = 0.02), and LDL (β = –9.99, SE = 3.40, Z = –2.94, p = 0.003) (Fig 4, S4 Table). For females, Thai elephants had higher insulin (β = –0.31, SE = 0.09, Z = –3.93, p = 0.0001), glucose (β = –12.00, SE = 2.55, Z = –4.71, p < 0.0001), HDL (β = –1.81, SE = 0.57, Z = –3.16, p = 0.0016), LDL (β = –2.91, SE = 1.26, Z = –2.30, p = 0.0213), and BCS (β = –1.006, SE = 0.12, Z = –8.40, p < 0.0001) than the females in Laos.

**Fig 5:**
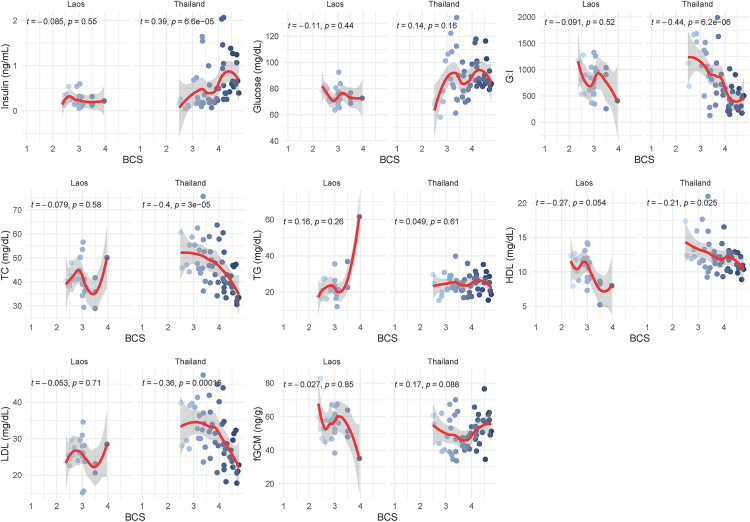
Relationships between body condition and lipid, metabolic, and fGCM factors separated by country. Kendall’s tau-b correlation between BCS, metabolic factors, lipid panel, and fGCM concentrations in captive Asian elephants in Laos (n = 27) and Thailand (n = 51). Abbreviations: τ = Kendall’s tau-b correlation coefficient; G:I = glucose to insulin ratio; TC = total cholesterol; TG = triglycerides; HDL = high density lipoproteins; LDL = low density lipoproteins; BCS = body condition score; fGCM = fecal glucocorticoid metabolites.

Correlations between BCS and physiological parameters had been investigated separately for each country and reported in Fig. 5. In Thailand, BCS showed positive correlations with insulin (τ = 0.39, p = 6.6e-05). Additionally, negative correlations were observed between BCS and G:I (τ = −0.44, p = 6.2e-06), TC (τ = −0.4, p = 3e-05), HDL (τ = −0.21, p = 0.02), and LDL (τ = −0.36, p = 0.00015). In contrast, among elephants in Laos, none of the parameters were significantly correlated with BCS.

## Discussion

This study provides the first direct comparison of metabolic, lipid, and stress profiles in captive Asian elephants managed under more naturalistic (Laos) versus intensive tourism (Thailand) systems. The goal was to contrast management practices between tourist camps in Thailand, where diets and activities are highly managed and elephants often participate in feeding and riding activities, and venues in Laos, where elephants spend more time free-roaming, socializing, and foraging naturally, with minimal supplemental feeding and tourist interactions. While the original goal of this research was to compare health and welfare biomarkers between these two disparate scenarios in relation to tourist activities, as it happens, the study began just as the COVID-19 pandemic closed all international travel, curtailing tourism in both countries. That provided a unique opportunity to focus specifically on management rather than human contact. Still, even with tourists taken out of the equation, a number of physiological differences were observed between countries. Elephants in Laos displayed more normal metabolic profiles and BCS compared to Thai counterparts, probably related to more natural foraging opportunities. Notably, despite the lack of tourist feeding, Thai elephants maintained higher BCS than their Lao counterparts, which were related to higher insulin, glucose, and lipid concentrations. A surprising finding was that elephants at the ECC had higher fGCM concentrations, which may reflect heightened physiological stimulation from new social opportunities, and disruptions in management practices caused by the pandemic. These results emphasize how management practices, specifically diet composition, exercise opportunities, and social and spatial freedom, can shape elephant health, advocating for more naturalistic approaches to enhance welfare.

### Metabolic profiles

Significant location and sex-based differences were observed in circulating glucose and insulin concentrations. Using comparable laboratory techniques, while most values were within normal ranges based on existing references [12,15,16,23,33], concentrations were overall higher in Thai elephants compared to those in Laos. The released elephants and those in the traditional management group within the Nam Phouy NPA had the lowest concentrations of metabolic markers (i.e., glucose, TC, HDL, and LDL), except for TG compared to all other groups, followed by elephants at the ECC. These metabolic differences may be related to diet. Elephants in Laos forage on a more natural/diverse diet throughout the year, especially the released/traditional management groups, and food supplements are not part of the diet. By contrast, the traditional diets of Thai elephants are highly managed, consisting of napier grass as the main fodder, with limited natural foraging opportunities, and feeding of high-calorie supplements like bananas and sugar cane by tourists. Even during the tourism ban, camp owners in Thailand continued to provide supplements, albeit in lower quantities [13], but apparently enough to continue having an effect on metabolic status. These results align with previous studies in black rhinoceros (*Diceros bicornis*) [34] and gorillas (*Gorilla gorilla gorilla*) [35], where higher insulin values were observed in captive-fed animals compared to free-roaming counterparts.

Metabolic profile results were associated with BCSs. In Thailand, higher BCS was found to be correlated with elevated insulin concentration, whereas no such associations were observed in the Lao elephants. This discrepancy can be explained by the distribution of BCSs within each group. In Thailand, a substantial proportion of tourist elephants were classified as overweight or obese: nearly 45% were assigned a BCS of 4, and almost 30% were given the highest score of 5 at the onset of the study [26]. By the end of the study, 23% of Thai elephants still had a BCS of 4. By contrast, only 3.33% of elephants in Laos had a BCS of 4, with none scoring 5 at any time during the study. With so few overweight or obese individuals present in Laos, it was not surprising that metabolic correlations with BCS were not detected.

There were no sex differences in BCS among the Lao elephants, whereas in Thailand, females tended to have higher, though not significant, BCSs corresponding to higher insulin concentrations and lower G:I compared to males. The latter agrees with studies before the COVID-19 pandemic in Thailand [15,16,33] and could be explained by the fact that females tend to be the preferred choice for interactions with tourists, like feeding, as they are more docile and predictable than males [36]. Although there was no tourist feeding in Thailand during the COVID-19 pandemic, female elephants started the study considerably fatter. Within the first 2 months of the study, 91.3% of females in Thailand had BCS = 4 or 5, while only 27.3% of the males did. In both countries, male elephants exhibited lower insulin concentrations than females, which could be due to dietary restrictions as a traditional practice to regulate body weight to better control the time and intensity of musth periods [16]. This phenomenon aligns with findings from a human study, in which insulin levels decreased as a result of time-restricted feeding [37]. However, further research is needed to explore and validate this hypothesis, as it could also suggest potential differences in sex-related metabolic or hormonal regulation.

### Lipid profiles and BCS

Location differences in relationships between BCS and lipids were also noted. Overall, BCS, TC, LDL, and HDL levels were higher in Thai compared to Lao elephants, which agrees with the observation that BCS remained relatively stable during the study in Laos, likely due to consistent food availability and exercise, even during the COVID-19 tourism ban. Sex differences were also apparent in the lipid panel, where males had higher TC and LDL than females in Thailand. However, these differences were not observed between male and female elephants in Laos. Previous studies in Sri Lankan elephants have reported higher TC in males than females, attributing these differences to physical activity, as exercise can reduce serum cholesterol [38]. However, in the present study, reported walking distances and hours spent on chains among the Thai population were similar between males (walking distance: 2.24 ± 0.21 km; chain hours: 23.70 ± 0.52h) and females (walking distance: 1.96 ± 0.10 km; chain hours: 22.49 ± 0.58h) [13,26], suggesting that other intrinsic or management-related factors may contribute to the observed metabolic differences.

Among Thai elephants, thinner individuals exhibited higher G:I,TC, HLD, and LDL concentrations. This phenomenon can be explained by the fact that animals with lower BCS have reduced fat reserves and may actively mobilize these stores to meet increased energy demands. During this metabolic process, fat deposits are broken down (lipolysis), releasing free fatty acids and other lipids into the bloodstream, which leads to elevated circulating lipid concentrations. This effect has been reported in pregnant and lactating dairy cows, where periods of negative energy balance, often due to increased metabolic demands, result in intensified fat mobilization and subsequently higher blood lipid levels such as non-esterified fatty acids [39]. In addition diets with low G:I had been shown to have benefits in terms of short-term glycemic control, weight, and adiposity [40]. In Laos, although the correlation was not significant, lower BCS was associated with higher HDL, a finding observed in humans, where an increase in physical activity was associated with an increase in HDL [41]. Lao elephants had regular walking routines that, in addition to naturalistic diets, could contribute to the increase in HDL concentrations and lower BCS. Therefore, these findings underscore the critical value of fostering active lifestyles within captive populations to support better health outcomes.

During the COVID-19 lockdown, Thai elephants experienced a marked reduction in physical activity, with some camps extending chaining periods for up to 48 contiguous hours, and chain lengths restricted to just 3 m during the day and 6 m at night [14,26]. In contrast, walking routines for elephants in Laos were unaffected by the absence of tourists. Elephants at the ECC continued to roam freely in the forests during the day, and at night were restrained with chains averaging 40 meters in length, allowing access to natural feeding grounds within an 80-meter diameter. Released elephants had unrestricted movement both day and night, while those under traditional management in the Nam Phouy NPA were only restrained occasionally.

In other species, moderate exercise is known to improve lipid homeostasis [42,43], while frequent, varied feedings, rather than a few large meals, can help suppress hunger and lower serum insulin [44]. As megaherbivores, Asian elephants are adapted to spend much of the day foraging on a wide variety of plant materials [45–47], and disruptions to this natural pattern, such as those observed in Thai tourist camps, are likely to contribute to the observed metabolic derangements. The Thai elephant’s low dietary diversity, reliance on sugar-rich supplements, and limited feeding frequency likely compounded the negative effects of inactivity, explaining the higher BCSs and less favorable lipid and metabolic panels compared to Lao counterparts, underscoring the protective effects of naturalistic management [8,23].

### fGCM and BCS

High BCSs have been linked to limited exercise, feeding of high-calorie treats, and higher fGCM concentrations in Thailand [15,16,33] and U.S. zoos [23,48]. By contrast, in the present study, the Lao population presented with lower BCSs and higher fGCM concentrations compared to the Thai population. Although the negative correlation between fGCM and BCS in the Lao elephants only approached significance, it suggests that as body condition decreased, fGCM concentrations tended to increase, a finding that has been reported for free-ranging Asian elephants in India [49]. Low BCSs could produce more physiological stress due to resource scarcity or other environmental challenges [49]. Thus, both low [50–53] and high [54–56] BCSs may be a stressful event for the body. These results highlight that both exercise and diet diversity are critical for maintaining healthy metabolic and lipid profiles in captive elephants, and that management practices mimicking natural conditions can promote better physiological outcomes.

### fGCM and seasonality and management factors

Seasonal peaks in fGCM were observed during the rainy (May – October) and winter (October-February) seasons in the Thai population, aligning with another Thai study conducted during the pandemic [26]. Elephants in Myanmar also exhibit higher fGCM concentrations during the monsoon season (May-September) [57]. In addition, these data also align with pre-pandemic findings in Thailand where fGCM concentrations peaked only during the winter during the high tourist season [15,16], suggesting that natural environmental factors could play an important role the in adrenal activity. Furthermore, since no seasonal variation was observed among the Lao population, it is possible that the elevated adrenal activity observed in Thai elephants is also influenced by management conditions. In Thailand, elephants are often restrained with short chains in non-naturalistic environments, which can increase stress responses during extreme weather events such as heavy rain or strong winds, as they have limited options to seek shelter, find comfort areas, or take refuge near other individuals. Providing elephants with more options to respond to adverse weather could be an important consideration for future management practices. In addition to seasonal variation, numerous other factors can influence GC concentrations. Aversive situations may elicit increases in concentrations, but they can also be increased by events considered to be neutral or pleasant [58]. Behaviorally, the ECC elephants rarely displayed behaviors commonly associated with stress, like stereotypies, whereas those are observed at high rates in Thailand tourist camps (57%) [59]. Rather, ECC elephants are more socially active, as both males and females have daily social interactions, especially compared to Thailand tourist camps. During the COVID-19 pandemic, the ECC initiated a social program for bulls to create all-male groups like those in the wild. Introducing new individuals can elicit stimulatory adrenal responses, especially during the initial months of the introduction [60–62]. During these social encounters, affiliative interactions were performed more frequently than aggressive and submissive behaviors and accounted for over 79.7% of the interactions (López Pérez et al., in prep). Thus, the elevated fGCM concentrations observed in ECC elephants, likely reflect the physiological arousal and adaptive stress response associated with increased social activity and the challenges of group restructuring, rather than distress. However, due to COVID-19 restrictions, several ECC mahouts chose to return to their hometowns, which may have compounded these effects. Studies demonstrate elephants respond more efficiently to familiar handlers, requiring weeks to months to acclimate to new caretakers [63,64]. Thus, higher fGCM at the ECC compared to Thailand and other Lao populations may reflect a multifactorial outcome of proactive social enrichment and pandemic-driven management disruptions, rather than a simple welfare deficit.

## Conclusions

This study provides the first comparative analysis of physiological health markers in captive Asian elephants managed under contrasting systems: forest-based naturalistic environments in Laos versus tourism-driven management in Thailand. The findings suggest that differences in diet, management practices, and daily activities play a role in shaping physiological health in captive elephants. Thai elephants exhibited higher glucose, insulin, and lipid levels, likely due to feeding low-diversity diets, supplemental foods rich in sugars, and limited exercise. In contrast, Lao elephants, especially those in the released and traditionally managed groups, had healthier metabolic and lipid profiles and generally maintained normal BCS (3–3.5), likely due to increased physical activity, social opportunities, and access to a diverse, natural diet. These differences persisted even during the COVID-19 tourism shutdown, emphasizing that baseline management practices, rather than tourist interaction alone, are drivers of health outcomes in these populations. Seasonal fluctuations in fGCM were observed among the Thai population, peaking during the rainy and winter seasons, suggesting that natural environmental factors could play an important role in adrenal activity, potentially compounded by the elephants’ limited ability to seek comfortable areas during adverse weather due to short restraints. In addition, the ECC elephants presented higher fGCM concentrations than in other locations, possibly due to changes in management (new mahouts and a novel social program for male elephants) that took place during the study period, rather than chronic stress.

These findings highlight the importance of managing elephants in natural environments, including daily exercise and diverse diets to enhance health and welfare. Future management plans should encourage tourist facilities to be located near forests, allowing elephants to forage and walk greater distances, and limit population sizes based on available space to permit free-roaming. When restraint is necessary, longer chains (20–30 m) should be used to allow greater movement, and elephants should be given the opportunity to interact with compatible companions throughout the day rather than being separated. Implementing these measures could substantially improve the long-term health and well-being of captive Asian elephants.

This study has several limitations that should be considered when interpreting findings. The small sample size of released and traditionally managed elephants in Laos may limit the statistical power and generalizability of comparisons across management types. The cross-sectional, observational design also restricts the ability to draw causal conclusions about the effects of management on specific health outcomes. Differences in laboratory conditions, including water quality, humidity, temperature, and technicians between Laos and Thailand could introduce measurement variability, despite our efforts to standardize assays. Body condition scoring, while using the same guidelines, remains a subjective assessment and may not fully capture differences in fat distribution or muscle mass. Finally, the unique circumstances of the COVID-19 pandemic, including changes in mahout staffing and management routines, may have further influenced the results in ways that are not representative of typical conditions. Now that pandemic restrictions are over, future studies are needed to compare elephants under more typical management conditions, allowing for better separation of the effects of routine practices, especially with respect to tourist activities, from those caused by pandemic-related disruptions.

## Supporting information

S1 Table GEE pairwise comparisons between locations. (DOCX)

S2 Table Seasonal effect on physiological parameters in Asian elephants. (DOCX)

S3 Table GEE pairwise seasonal comparisons between Lao and Thailand. (DOCX)

S4. Table Sex effects on physiological parameters in Asian elephants. (DOCX)
