# Supplementary material for: Metabolic profiles of captive Asian elephants (Elephas maximus) in Lao PDR and Thailand
Source: PLoS One. 2025 Dec 17;20(12):e0334550. doi: 10.1371/journal.pone.0334550 (PMC12711091; doi:10.1371/journal.pone.0334550)
Supplement: S1 Table — (DOCX) [file pone.0334550.s001.docx]

S1 Table. Pairwise comparisons of GEE model results for metabolic factors, lipids, BCS, and fGCM measures in captive Asian elephants from the released and traditionally managed group (n = 8), the Elephant Conservation Center in Laos (n = 19), and tourist camps in Thailand (n = 51).

| **Pairwise comparisons** | **β (Estimate)** | **SE** | **Z** | **p-value** |
| --- | --- | --- | --- | --- |
| **Insulin (ng/mL)** |  |  |  |  |
| ECC vs. Released/traditional management | 0.130 | 0.062 | 2.100 | 0.107 |
| ECC vs. Thailand tourist camps | -0.282 | 0.080 | -3.520 | 0.001 |
| Released/traditional management vs. Thailand tourist camps | -0.412 | 0.081 | -5.080 | <0.001 |
| **Glucose (mg/dL)** |  |  |  |  |
| ECC vs. Released/traditional management | 4.000 | 2.180 | 1.830 | 0.200 |
| ECC vs. Thailand tourist camps | -11.700 | 2.750 | -4.250 | 0.0001 |
| Released/traditional management vs. Thailand tourist camps | -15.700 | 2.790 | -5.620 | <0.001 |
| **G:I** |  |  |  |  |
| ECC vs. Released/traditional management | 100.100 | 110.100 | 0.908 | 1.000 |
| ECC vs. Thailand tourist camps | 17.800 | 91.600 | 0.194 | 1.000 |
| Released/traditional management vs. Thailand tourist camps | -82.300 | 102.700 | -0.801 | 1.000 |
| **TC (mg/dL)** |  |  |  |  |
| ECC vs. Released/traditional management | 2.630 | 2.320 | 1.134 | 0.771 |
| ECC vs. Thailand tourist camps | -3.660 | 2.230 | -1.643 | 0.301 |
| Released/traditional management vs. Thailand tourist camps | -6.290 | 2.410 | -2.614 | 0.027 |
| **TG (mg/dL)** |  |  |  |  |
| ECC vs. Released/traditional management | -5.010 | 3.970 | -1.260 | 0.623 |
| ECC vs. Thailand tourist camps | -2.530 | 1.480 | -1.750 | 0.265 |
| Released/traditional management vs. Thailand tourist camps | 2.480 | 4.300 | 0.576 | 1.000 |
| **HDL (mg/dL)** |  |  |  |  |
| ECC vs. Released/traditional management | 1.550 | 0.726 | 2.130 | 0.098 |
| ECC vs. Thailand tourist camps | -1.590 | 0.593 | -2.680 | 0.022 |
| Released/traditional management vs. Thailand tourist camps | -3.140 | 0.713 | -4.410 | <0.0001 |
| **LDL (mg/dL)** |  |  |  |  |
| ECC vs. Released/traditional management | 2.030 | 1.760 | 1.150 | 0.747 |
| ECC vs. Thailand tourist camps | -4.340 | 1.590 | -0.273 | 0.019 |
| Released/traditional management vs. Thailand tourist camps | -6.370 | 1.680 | -3.800 | <0.0001 |
| **BCS (mg/dL)** |  |  |  |  |
| ECC vs. Released/traditional management | -0.379 | 0.079 | -4.770 | <0.0001 |
| ECC vs. Thailand tourist camps | -0.881 | 0.101 | -8.720 | <0.0001 |
| Released/traditional management vs. Thailand tourist camps | -0.502 | 0.109 | -4.458 | <0.0001 |
| **fGCM (ng/g)** |  |  |  |  |
| ECC vs. Released/traditional management | 7.870 | 3.270 | 2.403 | 0.049 |
| ECC vs. Thailand tourist camps | 7.640 | 2.620 | 2.919 | 0.011 |
| Released/traditional management vs. Thailand tourist camps | -0.230 | 2.750 | -0.083 | 1.000 |

Released/traditional management: four from the Elephant Conservation Center (ECC) that were part of a soft-release program in 2019; four managed under traditional conditions.

Abbreviations: G:I = glucose to insulin ratio; TC = total cholesterol; TG = triglycerides, LDL = low density lipoproteins; HDL = high density lipoproteins; BCS = body condition score; fGCM = fecal glucocorticoid metabolites.
