# Supplementary material for: Metabolic profiles of captive Asian elephants (Elephas maximus) in Lao PDR and Thailand
Source: PLoS One. 2025 Dec 17;20(12):e0334550. doi: 10.1371/journal.pone.0334550 (PMC12711091; doi:10.1371/journal.pone.0334550)
Supplement: S2 Table — (DOCX) [file pone.0334550.s002.docx]

S2 Table. Seasonal effects on physiological parameters in Asian elephants. Mean ± SEM values for metabolic biomarkers, lipids, body condition score, and fecal glucocorticoid metabolite concentrations in Asian elephants in Laos (n = 27) and tourist camps in Thailand (n=51).

|  | Laos | | | Thailand | | |
| --- | --- | --- | --- | --- | --- | --- |
| Parameters | **Summer** | **Rainy** | **Winter** | **Summer** | **Rainy** | **Winter** |
| Insulin (ng/mL) | 0.28 ± 0.03^a,x^ | 0.19 ± 0.02^a,x^ | 0.28 ± 0.03^a,x^ | 0.62 ± 0.07^a,y^ | 0.42 ± 0.05^a,y^ | 0.75 ± 0.10^a,y^ |
| Glucose (mg/dL) | 75.50 ± 1.28^a,x^ | 73.63 ± 1.23^a,x^ | 76.55 ± 1.30^a,x^ | 91.15 ± 1.72^a,y^ | 89.90 ± 1.62^a,y^ | 84.98 ± 1.82^a,y^ |
| G:I | 735.85 ± 45.99^a,x^ | 898.02 ± 57.19^a,x^ | 736.28 ± 52.81^a,x^ | 724.00 ± 64.41^a,x^ | 782.76 ± 56.05^a,x^ | 702.76 ± 63.11^a,x^ |
| TC  (mg/dL) | 42.53 ± 0.66^a,x^ | 40.82 ± 0.84^a,x^ | 41.79 ± 0.65^a,x^ | 45.67 ± 1.14^a,y^ | 46.28 ± 0.89^a,x^ | 46.69 ± 0.92^a,y^ |
| TG  (mg/dL) | 23.08 ± 1.10^a,x^ | 25.28 ± 0.99^a,x^ | 22.12 ± 0.94^a,x^ | 27.01 ± 1.08^a,x^ | 25.56 ± 0.76^a,x^ | 21.68 ± 0.74^a,x^ |
| HDL  (mg/dL) | 10.81 ± 0.27^b,x^ | 10.02 ± 0.26^ab,x^ | 10.75 ± 0.25^a,x^ | 12.17 ± 0.32^a,y^ | 12.20 ± 0.24^a,y^ | 12.37 ± 0.23^a,y^ |
| LDL  (mg/dL) | 26.10 ± 0.50^a,x^ | 24.76 ± 0.53^a,x^ | 25.62 ± 0.46^a,x^ | 29.64 ± 0.79^a,y^ | 32.12 ± 0.65^a.y^ | 29.89 ± 0.65^a,y^ |
| BCS  (1-5) | 2.89 ± 0.03^a,x^ | 2.97 ± 0.03^a,x^ | 2.92 ± 0.03^a,x^ | 3.82 ± 0.05^a,y^ | 3.84 ± 0.05^a,y^ | 3.78 ± 0.06^a,y^ |
| fGCM  (ng/g) | 56.17 ± 1.79^a,^**^y^** | 57.52 ± 1.67^a,^**^x^** | 54.53 ± 2.04^a,^**^x^** | 42.93 ± 1.31^a,^**^x^** | 52.55 ± 1.51^b,^**^x^** | 53.36 ± 1.49^b,^**^x^** |

Abbreviations: G:I = glucose to insulin ratio; TC = total cholesterol; HDL = high density lipoproteins; LDL = low density lipoproteins; BCS = body condition score; fGCM = fecal glucocorticoid metabolites.

Summer = 16 February-15 May; Rainy = 16 May-15 October; Winter = 16 October-15 February.

^a,b^Different letters between columns indicate significant differences for each variable across Summer, Rainy, and Winter seasons within each country when subjected to GEE model (p < 0.05). Superscript letters (a, b) were assigned in order of concentration, with a representing the lowest concentration.

^x,y^Different letters between columns indicate significant differences for each variable across Summer, Rainy, and Winter seasons between countries when subjected to GEE model (p < 0.05). Superscript letters (x,y) were assigned in order of concentration, with x representing the lowest concentration.
