# Supplementary material for: Metabolic profiles of captive Asian elephants (Elephas maximus) in Lao PDR and Thailand
Source: PLoS One. 2025 Dec 17;20(12):e0334550. doi: 10.1371/journal.pone.0334550 (PMC12711091; doi:10.1371/journal.pone.0334550)
Supplement: S3 Table — (DOCX) [file pone.0334550.s003.docx]

S3 Table. Seasonal pairwise comparisons of GEE model results for metabolic factors, lipids, body condition score, and fecal glucocorticoid metabolite concentrations in captive Asian elephants in Laos (n = 27) and Thailand (n = 51).

| **Comparison** | **β (Estimate)** | **SE** | **Z** | **p-value** |
| --- | --- | --- | --- | --- |
|  |  |  |  |  |
| **Insulin (ng/mL)** |  |  |  |  |
| Rainy (Laos vs Thailand) | -0.194 | 0.072 | -2.690 | 0.007 |
| Summer (Laos vs Thailand) | -0.359 | 0.104 | -3.440 | 0.0006 |
| Winter (Laos vs Thailand) | -0.410 | 0.132 | -3.110 | 0.002 |
| **Glucose (mg/dL)** |  |  |  |  |
| Rainy (Laos vs Thailand) | -10.400 | 4.300 | -2.420 | 0.015 |
| Summer (Laos vs Thailand) | -14.900 | 3.470 | -4.310 | <0.0001 |
| Winter (Laos vs Thailand) | -11.900 | 3.230 | -3.690 | 0.0002 |
| **G:I** |  |  |  |  |
| Rainy (Laos vs Thailand) | 8.600 | 107.000 | 0.080 | 0.936 |
| Summer (Laos vs Thailand) | -67.800 | 106.000 | -0.641 | 0.521 |
| Winter (Laos vs Thailand) | 38.000 | 109.000 | 0.348 | 0.728 |
| **TC (mg/dL)** |  |  |  |  |
| Rainy (Laos vs Thailand) | -2.120 | 2.680 | -0.791 | 0.429 |
| Summer (Laos vs Thailand) | -5.230 | 2.060 | -2.537 | 0.011 |
| Winter (Laos vs Thailand) | -5.040 | 2.340 | -2.155 | 0.031 |
| **TG (mg/dL)** |  |  |  |  |
| Rainy (Laos vs Thailand) | 1.700 | 2.270 | 0.746 | 0.456 |
| Summer (Laos vs Thailand) | -2.640 | 2.330 | -1.130 | 0.258 |
| Winter (Laos vs Thailand) | -1.910 | 2.100 | -0.911 | 0.362 |
| **HDL (mg/dL)** |  |  |  |  |
| Rainy (Laos vs Thailand) | -2.370 | 0.711 | -3.330 | 0.0009 |
| Summer (Laos vs Thailand) | -1.510 | 0.555 | -2.720 | 0.007 |
| Winter (Laos vs Thailand) | -2.790 | 0.635 | -4.390 | <0.0001 |
| **LDL (mg/dL)** |  |  |  |  |
| Rainy (Laos vs Thailand) | -4.890 | 1.820 | -2.690 | 0.007 |
| Summer (Laos vs Thailand) | -4.720 | 1.460 | -3.240 | 0.001 |
| Winter (Laos vs Thailand) | -5.540 | 1.660 | -3.350 | 0.0008 |

| **Comparison** | **β (Estimate)** | **SE** | **Z** | **p-value** |
| --- | --- | --- | --- | --- |
| **BCS (1-5)** |  |  |  |  |
| Rainy (Laos vs Thailand) | -0.660 | 0.123 | -5.350 | <0.0001 |
| Summer (Laos vs Thailand) | -0.834 | 0.103 | -8.080 | <0.0001 |
| Winter (Laos vs Thailand) | -0.700 | 0.115 | -6.100 | <0.0001 |
| **fGCM (ng/g)** |  |  |  |  |
| Rainy (Laos vs Thailand) | -3.730 | 3.250 | -1.150 | 0.251 |
| Summer (Laos vs Thailand) | -11.830 | 2.480 | -4.770 | <0.0001 |
| Winter (Laos vs Thailand) | -0.530 | 3.430 | -0.150 | 0.877 |

Abbreviations: G:I = glucose to insulin ratio; TC = total cholesterol; TG = triglycerides; LDL = low density lipoproteins; HDL = high density lipoproteins; BCS = body condition score; fGCM = fecal glucocorticoid metabolites.

Summer = 16 February-15 May; Rainy = 16 May-15 October; Winter = 16 October-15 February.
