# Supplementary material for: Metabolic profiles of captive Asian elephants (Elephas maximus) in Lao PDR and Thailand
Source: PLoS One. 2025 Dec 17;20(12):e0334550. doi: 10.1371/journal.pone.0334550 (PMC12711091; doi:10.1371/journal.pone.0334550)
Supplement: S4. Table — (DOCX) [file pone.0334550.s004.docx]

S4 Table. Sex effects on physiological parameters in Asian elephants. Mean ± SEM values for metabolic biomarkers, lipids, body condition score, and fecal glucocorticoid metabolite concentrations in Asian elephants in Laos (n = 27) and tourist camps in Thailand (n=51).

|  | Laos |  |  | Thailand |  |
| --- | --- | --- | --- | --- | --- |
| Parameters | **Mean** | **Range** | **Parameters** | **Mean** | **Range** |
| Insulin (ng/mL) |  |  | **Insulin (ng/mL)** |  |  |
| Male | 0.24 ± 0.03^a,x^ | 0.02 - 1.90 | Male | 0.44 ± 0.08^a,x^ | 0.02 - 5.96 |
| Female | 0.25 ± 0.02^a,x^ | 0.03 - 3.26 | Female | 0.61 ± 0.05^a,y^ | 0.02 - 9.77 |
| Glucose (mg/dL) |  |  | **Glucose (mg/dL)** |  |  |
| Male | 75.56 ± 1.50^a,x^ | 32.00 - 161.00 | Male | 91.12 ± 2.78^a,y^ | 36.00 - 203.00 |
| Female | 74.95 ± 0.78^a,x^ | 46.00 - 143.00 | Female | 88.17 ± 1.03^a,y^ | 23.00 - 193.00 |
| G:I |  |  | **G:I** |  |  |
| Male | 971.43 ± 58.43^b,x^ | 67.32 - 3840.00 | Male | 1113.23 ± 95.63^b,x^ | 27.00 - 3800.00 |
| Female | 693.33 ± 32.97^a,x^ | 48.63 - 2530.44 | Female | 652.27 ± 36.03^a,x^ | 10.95 - 3960.00 |
| TC (mg/dL) |  |  | **TC (mg/dL)** |  |  |
| Male | 41.90 ± 0.73^a,x^ | 21.00 - 67.50 | Male | 52.00 ± 1.46^b,y^ | 23.00 - 124.00 |
| Female | 41.54 ± 0.51^a,x^ | 19.33 - 78.00 | Female | 44.60 ± 0.59^a,x^ | 19.00 - 114.00 |
| TG (mg/dL) |  |  | **TG (mg/dL)** |  |  |
| Male | 20.79 ± 0.58^a,x^ | 8.00 - 44.00 | Male | 25.10 ± 1.27^a,x^ | 2.00 - 90.00 |
| Female | 25.12 ± 0.84^a,x^ | 4.00 - 122.00 | Female | 24.73 ± 0.54^a,x^ | 2.00 - 100.00 |
| HDL (mg/dL) |  |  | **HDL (mg/dL)** |  |  |
| Male | 11.38 ± 0.25^a,x^ | 5.00 - 20.50 | Male | 13.58 ± 0.42^a,x^ | 6.40 - 33.20 |
| Female | 10.02 ± 0.18^a,x^ | 4.00 - 20.50 | Female | 11.91 ± 0.15^a,x^ | 4.80 - 33.70 |
| LDL (mg/dL) |  |  | **LDL (mg/dL)** |  |  |
| Male | 25.21 ± 0.57^a,x^ | 9.00 - 44.50 | Male | 34.58 ± 0.95^b,y^ | 10.30 - 75.20 |
| Female | 25.62 ± 0.32^a,x^ | 10.67 - 43.00 | Female | 29.71 ± 0.43^a,y^ | 11.60 - 80.80 |
| BCS (1 - 5) |  |  | **BCS (1 - 5)** |  |  |
| Male | 2.88 ± 0.03^a,x^ | 2.00 - 3.50 | Male | 3.02 ± 0.06^a,x^ | 2.00 - 5.00 |
| Female | 2.96 ± 0.02^a,x^ | 2.00 - 4.00 | Female | 4.02 ± 0.03^b,y^ | 1.50 - 5.00 |
| fGCM (ng/g) |  |  | **fGCM (ng/g)** |  |  |
| Male | 60.44 ± 1.94^a,y^ | 22.91 - 161.17 | Male | 52.37 ± 1.99^a,x^ | 17.42 - 127.43 |
| Female | 53.42 ± 1.21^a,x^ | 4.94 - 145.12 | Female | 49.86 ± 0.98^a,x^ | 16.94 - 175.96 |

^a,b,^Different letters between columns indicate significant differences for each variable within columns when subjected to GEE model (p < 0.05). Superscript letters (a, b) were assigned in order of concentration, with a representing the lowest concentration.

^x,y^Different letters between columns indicate significant differences for each sex between countries when subjected to GEE model (p < 0.05). Superscript letters (x,y) were assigned in order of concentration, with x representing the lowest concentration.

Abbreviations: G:I = glucose to insulin ratio; TC = total cholesterol; TG = triglycerides; HDL = high density lipoproteins; LDL = low density lipoproteins; BCS = body condition score; fGCM = fecal glucocorticoid metabolites.
